# Cortical lobar volume reductions associated with homocysteine-related subcortical brain atrophy and poorer cognition in healthy aging

**DOI:** 10.3389/fnagi.2024.1406394

**Published:** 2024-08-07

**Authors:** Hyun Song, Pradyumna K. Bharadwaj, David A. Raichlen, Christian G. Habeck, Matthew D. Grilli, Matthew J. Huentelman, Georg A. Hishaw, Theodore P. Trouard, Gene E. Alexander

**Affiliations:** ^1^Department of Psychology, University of Arizona, Tucson, AZ, United States; ^2^Evelyn F. McKnight Brain Institute, University of Arizona, Tucson, AZ, United States; ^3^Human and Evolutionary Biology Section, Department of Biological Sciences, University of Southern California, Los Angeles, CA, United States; ^4^Cognitive Neuroscience Division, Department of Neurology and Taub Institute, Columbia University, New York, NY, United States; ^5^Department of Neurology, University of Arizona, Tucson, AZ, United States; ^6^Neurogenomics Division, The Translational Genomics Research Institute (TGen), Phoenix, AZ, United States; ^7^Arizona Alzheimer's Consortium, Phoenix, AZ, United States; ^8^Department of Biomedical Engineering, University of Arizona, Tucson, AZ, United States; ^9^Department of Psychiatry, University of Arizona, Tucson, AZ, United States; ^10^Neuroscience and Physiological Sciences Graduate Interdisciplinary Programs, University of Arizona, Tucson, AZ, United States

**Keywords:** cognitive aging, vascular risk, scaled subprofile model, white matter hyperintensity, gray matter atrophy

## Abstract

Homocysteine (Hcy) is a cardiovascular risk factor implicated in cognitive impairment and cerebrovascular disease but has also been associated with Alzheimer’s disease. In 160 healthy older adults (mean age = 69.66 ± 9.95 years), we sought to investigate the association of cortical brain volume with white matter hyperintensity (WMH) burden and a previously identified Hcy-related multivariate network pattern showing reductions in subcortical gray matter (SGM) volumes of hippocampus and nucleus accumbens with relative preservation of basal ganglia. We additionally evaluated the potential role of these brain imaging markers as a series of mediators in a vascular brain pathway leading to age-related cognitive dysfunction in healthy aging. We found reductions in parietal lobar gray matter associated with the Hcy-SGM pattern, which was further associated with WMH burden. Mediation analyses revealed that slowed processing speed related to aging, but not executive functioning or memory, was mediated sequentially through increased WMH lesion volume, greater Hcy-SGM pattern expression, and then smaller parietal lobe volume. Together, these findings suggest that volume reductions in parietal gray matter associated with a pattern of Hcy-related SGM volume differences may be indicative of slowed processing speed in cognitive aging, potentially linking cardiovascular risk to an important aspect of cognitive dysfunction in healthy aging.

## 1 Introduction

As a sulfur amino acid, homocysteine (Hcy) is produced via the metabolism of the dietary essential amino acid methionine whose disturbances related to increasing age may lead to excess plasma concentrations of Hcy (Hankey and Eikelboom, 2001). Raised levels of Hcy have been recognized as a cardiovascular risk factor in healthy aging associated with age-related cognitive dysfunction (Prins et al., 2002; Dufouil et al., 2003), but have also been associated with greater risk for dementia related to cerebrovascular disease (CVD; Hankey and Eikelboom, 2001), as well as Alzheimer’s disease (AD; Seshadri et al., 2002). Hcy is pro-atherothrombotic (Hankey and Eikelboom, 2001) and higher levels of Hcy have been associated with greater white matter hyperintensity (WMH) burden on magnetic resonance imaging (MRI) in studies of middle-to-older-aged adults (Wright et al., 2005; Raz et al., 2012). The pathway by which elevated plasma Hcy levels contribute to cognitive aging and the risk for both CVD and AD, however, remains to be fully elucidated.

In a prior study (Song et al., 2023), we used multivariate network covariance analysis with the Scaled Subprofile Model (SSM; Alexander and Moeller, 1994) to identify a network pattern of Hcy-related subcortical gray matter (SGM) volumes, reflecting regional covariance in subcortical brain areas. This pattern indicated associations between higher plasma Hcy levels and volume reductions in both hippocampus and nucleus accumbens, key subcortical brain structures often implicated in the development of CVD and AD in aging (Walhovd et al., 2005; Habes et al., 2016; Pini et al., 2016; Werden et al., 2017; Morys et al., 2021), with relative preservation of basal ganglia volumes. Furthermore, we showed that increased WMH burden during aging predicted greater expression of the multivariate Hcy-related SGM volume pattern, which together sequentially mediated diminished processing speed, but not with measures of memory or executive function, in healthy older adults (Song et al., 2023).

While these findings suggest that the SSM Hcy-SGM pattern may provide an important early brain-based indicator of cognitive aging, reflecting a potential link between cardiovascular risk, CVD, and an aspect of cognitive dysfunction, our previous study was limited to investigating the volumes of subcortical brain structures related to plasma Hcy levels. Vascular white matter lesions, as well as plasma Hcy, have also been shown to contribute to alterations in cortical brain structures (Seshadri et al., 2008; Raji et al., 2012; Habes et al., 2016; Lambert et al., 2016; Tan et al., 2018). Structural MRI studies have suggested reduced gray matter volume (GMV) or thickness associated with higher Hcy levels (Seshadri et al., 2008; Tan et al., 2018) and greater WMH volume (Raji et al., 2012; Lambert et al., 2015; Habes et al., 2016) in brain regions often affected in aging and AD, including cortical regions of the frontal, temporal, and parietal lobes (Resnick et al., 2003; Alexander et al., 2006; Pini et al., 2016). Furthermore, reduced cortical gray matter has been linked to age-related decrements in processing speed, executive functioning, and memory (Cardenas et al., 2011; Mungas et al., 2018).

Importantly, studies have yet to investigate whether and how cortical brain volumes relate to Hcy-related subcortical volumetric differences and WMH lesion load. Investigating whether age-related differences in cortical brain volumes are mediated by Hcy-SGM pattern differences associated with WMH lesions to influence cognitive aging may advance our understanding of the structural brain-based effects of Hcy. Such efforts may help further elucidate a possible pathway by which a common peripheral blood marker of cardiovascular risk is associated with a combination of brain-based cortical and subcortical impacts that can contribute to cognitive dysfunction in healthy aging.

The present study built upon our previous work (Song et al., 2023) in the same cohort of healthy older adults to investigate whether and how lobar regions of cortical brain volume are associated with our previously identified regional network covariance pattern that showed decreased SGM volumes of both the hippocampus and nucleus accumbens related to elevated plasma Hcy, as well as WMH burden. We additionally investigated the potential role of these cortical and subcortical brain imaging markers as a series of mediators to evaluate a novel vascular risk pathway to influence cognitive dysfunction in healthy aging. To test associations between the structural brain-based effects of Hcy and cognition separate from overall cardiovascular health, we adjusted mediation models for cardiorespiratory fitness measured by maximal oxygen uptake (VO_2_max) obtained during a treadmill graded exercise test (GXT), as well as other common AD and CVD risk factors (i.e., apolipoprotein E [APOE] ε4 status, hypertension status, smoking history, and additionally vitamin B12 levels). We hypothesized that cortical volume reductions in regions often implicated in aging and the risk for CVD and AD, including frontal, parietal, and temporal lobe areas, would be predicted by the Hcy-SGM pattern. We also hypothesized that greater WMH lesion volume related to aging would predict greater expression of the Hcy-SGM pattern, followed by cortical brain atrophy, which in turn would be associated with diminished processing speed, executive functions, and memory in healthy older adults.

## 2 Materials and methods

### 2.1 Participants

Participants were the same cohort as reported in our prior study (Song et al., 2023), which included 160 community-dwelling healthy, cognitively unimpaired older adults, 50–89 years of age (mean age = 69.66 ± 9.95 years, 53.8% women, 95.0% White individuals with 5.0% of those self-identifying as Hispanic/Latinx). Participant characteristics have been previously reported in detail (Song et al., 2023) and are shown in Table 1. All participants provided written consent after they were informed about the study procedures and possible risks of participation. The study was reviewed and approved by the Institutional Review Board at the University of Arizona.

**Table 1:** Characteristics of the study sample.

Variable	Mean *± SD*
Age (years)	69.66 *±* 9.95
Sex (female/male)	86/74
Education (years)	15.98 *±* 2.57
MMSE score	29.01 *±* 1.25
Total WMH volume (mL)	6.57 *±* 10.53
Frontal GMV (mL)	164.83 *±* 15.04
Temporal GMV (mL)	96.83 *±* 9.62
Parietal GMV (mL)	115.52 *±* 11.19
Occipital GMV (mL)	44.91 *±* 5.46
TMT-A (seconds)	32.18 *±* 10.37
TMT-B (seconds)	76.14 *±* 30.50
SRT CLTR (words)	64.62 *±* 37.07

*SD* represents standard deviation. Details of participant characteristics have been reported previously (Song et al., 2023).

CLTR, consistent long-term retrieval; GMV, gray matter volume; MMSE, Mini-Mental Status Exam; SRT, Buschke Selective Reminding Test; TMT, Trail Making Test; WMH, white matter hyperintensity.

### 2.2 Laboratory assessments

Fasting blood samples drawn during the imaging visit were prepared for analysis and plasma total Hcy was processed as described previously (Song et al., 2023). In brief, total Hcy was determined in plasma by the fluorescence polarization immunoassay method on the Axsym analyzer or by the chemiluminescence microparticle immunoassay method on the ci8200 analyzer (Abbott Laboratories, Chicago, IL). Validation between these two analyzers was conducted and confirmed with a Deming regression coefficient of >0.999.

Serum vitamin B12 was determined by enhanced chemiluminescence immunoassay on the Vitros ECi Immunodiagnostic System (Ortho Clinical Diagnostics, Raritan, NJ).

APOE genotype was determined as previously described (Van Etten et al., 2021). In brief, extracted DNA was assayed using restriction fragment length polymorphism analysis. DNA amplification by polymerase chain reaction was followed by digestion with HhaI restriction enzyme, and agarose gel analysis (Addya et al., 1997).

### 2.3 Cardiorespiratory fitness assessment

Oxygen uptake was measured during a maximal treadmill GXT using a modified Naughton protocol (Berry et al., 1996) with standard techniques of open-circuit spirometry as previously described (Song et al., 2023). Achievement of VO_2_max, the standard measure of cardiorespiratory fitness, was considered as the attainment of at least two of the following three criteria: (1) a plateau in oxygen consumption with an increase in workload; (2) a respiratory exchange ratio of ≥1.1; and (3) a heart rate within 10 beats/min of the age-predicted maximum rate (ACSM, 2000).

### 2.4 Magnetic resonance imaging

Structural MRI images were acquired on a General Electric 3.0 Tesla scanner (HD Signa Excite, Milwaukee, WI). We obtained volumetric T1-weighted images using a spoiled gradient echo sequence with the following parameters: TR = 5.3 ms, TE = 2.0 ms, TI = 500 ms, slice thickness = 1 mm, flip angle = 15°, matrix = 256 × 256, field of view = 256 mm. We obtained fluid attenuated inverse recovery (FLAIR) T2-weighted scans with the following parameters: TR = 11,000 ms, TE = 120 ms, TI = 2,250 ms, slice thickness = 2.6 mm, flip angle = 90°, matrix = 256 × 256, field of view = 256 mm.

To process T1-weighted MRI scans, we used the default *recon-all* pipeline of FreeSurfer v5.3.^1^ Details of the processing stages for the automated segmentation of subcortical and cortical brain structures have been described elsewhere (Fischl et al., 2002, 2004). In brief, the FreeSurfer processing pipeline included non-brain tissue removal, Talairach transformation, gray/white matter segmentation, intensity normalization, tessellation of gray/white matter boundaries, automated correction of topological defects, and surface deformation. The processing stream provided GMVs of 34 bilateral cortical regions of interest (ROIs) according to the Desikan-Killiany atlas (Desikan et al., 2006), as well as seven subcortical brain structures for each hemisphere (Song et al., 2023). Volumes in the relevant ROIs that comprise each lobe were combined to derive lobar GMVs for the four major lobes (Desikan et al., 2006; Seshadri et al., 2008; Klein and Tourville, 2012; Chao et al., 2014; Dong et al., 2015).

Total WMH volume was automatically segmented using a combination of T1 and T2 FLAIR images with the lesion segmentation toolbox (Schmidt et al., 2012) implemented in Statistical Parametric Mapping (SPM12; Wellcome Trust Center for Neuroimaging, London, United Kingdom), using the lesion growth algorithm (LGA) approach. The processing steps involved in the segmentation of WMH in this healthy older adult cohort have been previously described in detail (Franchetti et al., 2020; Van Etten et al., 2021). Briefly, reference WMH maps were manually segmented in a subset of 35 participants using ITK-SNAP^2^ and underwent consensus review by expert raters. Mean spatial overlap between the LGA-generated WMH maps and the manually segmented reference WMH maps was computed across a range of optimization parameter kappa values (0.05–1.00) to determine an optimal threshold of 0.35 at which lesion probability maps for all study participants were generated. Total WMH volume was calculated as the sum of voxel volumes and was log-transformed. Estimates of total intracranial volume (TIV) were also obtained in native brain space from each T1 image using SPM12 (Alexander et al., 2012a).

### 2.5 Neuropsychological outcomes

We used measures of cognitive domains that are particularly vulnerable to the effects of aging: processing speed, executive functioning, and verbal memory (Alexander et al., 2012b), focusing on three selected measures obtained from the Trail Making Test (TMT; Reitan, 1958), Parts A and B and the 12-word, 12-trial version of the Buschke Selective Reminding Test (SRT; Buschke, 1973) as in our previous study (Song et al., 2023). Such selected cognitive measures included the total time to completion of TMT-A (after log-transformation), which provides a measure of visuomotor processing speed that requires participants to draw lines connecting numbered circles randomly distributed on a sheet of paper in numerical order (Salthouse et al., 2000; Sánchez-Cubillo et al., 2009; Jacobs et al., 2013; Ferris et al., 2022). TMT-B is a timed task that involves drawing lines to connect numbered and lettered circles in alternating sequences. We used the standardized residual values of TMT-B, obtained by statistically removing the processing speed performance on TMT-A using raw scores, as a measure of executive functioning (Salthouse et al., 2000; Sánchez-Cubillo et al., 2009; Jacobs et al., 2013; Ferris et al., 2022). We also included the number of words consistently recalled on at least three succeeding trials, scored as consistent long-term retrieval (CLTR), on the SRT as a measure of verbal memory (Buschke, 1973). Despite non-significant associations of the Hcy-SGM pattern with executive functions or memory observed in our prior work, we included the same selected measures of these cognitive domains to assess their potential associations with cortical gray matter volumes (Cardenas et al., 2011; Mungas et al., 2018) while maintaining comparability in evaluating our mediation models across studies.

### 2.6 Statistical analyses

Details of multivariate SSM analysis to identify subcortical regional covariance related to total plasma Hcy have been described previously (Song et al., 2023). In brief, following a modified principal component analysis, we chose the first set of sequential components as the best set of SSM component patterns predicting plasma total Hcy based on the lowest value of Akaike information criteria (Burnham and Anderson, 2002). A bootstrap resampling procedure was applied with 10,000 iterations to the SSM analysis to obtain reliability estimates at each volume of subcortical brain structures for the observed pattern weights related to Hcy (Efron and Tibshirani, 1994; Habeck et al., 2005; Alexander et al., 2012a). In our previous work, univariate associations of Hcy with individual subcortical brain volumes were tested as a follow-up to the multivariate SSM analysis to clarify how each of the regions in the pattern was associated with Hcy. The follow-up analyses showed that higher Hcy levels were significantly related to smaller hippocampal and nucleus accumbens volumes but were not significantly related to basal ganglia volumes. These findings suggested that the multivariate Hcy-related SGM pattern was mainly characterized by volume reductions in hippocampus and nucleus accumbens with increasing Hcy levels, while the covarying pattern increases in basal ganglia volumes may have reflected relative preservation of these subcortical brain structures with greater Hcy (Song et al., 2023).

Using our previously established Hcy-SGM covariance pattern, we performed block-wise multiple regression analyses to test the prediction of lobar GMVs by the multivariate Hcy-related SGM pattern. We first adjusted for TIV (Block 1) and then age, sex, and education (Block 2) to investigate the pattern’s association with cortical lobar brain volumes, distinct from differences in head size and additionally demographic factors (Mungas et al., 2018) while correcting for multiple comparisons with false discovery rate (FDR; Benjamini and Hochberg, 1995; Benjamini, 2010). As a follow-up, these analyses were repeated for left and right lobar GMVs separately to evaluate whether findings were consistent across hemispheres.

Significant FDR-corrected associations between lobar GMVs and the Hcy-SGM pattern were followed by serial mediation model analyses where we evaluated their relationships with WMH lesions. In these serial mediation models, we tested age as a predictor and lobar cortical GMVs separately as dependent variables, and total WMH volume and the Hcy-SGM pattern sequentially as mediators. We initially included TIV, sex, and education as covariates to account for their potential association with white matter lesion load and gray matter volume (Wright et al., 2005; Kern et al., 2017). We subsequently entered APOE ε4 status, hypertension status, smoking history, VO_2_max, and additionally vitamin B12 levels as added covariates to account for their relation to WMH lesions and volume reductions in gray matter as CVD and AD risk factors (Hankey and Eikelboom, 2001; Wright et al., 2005; Raji et al., 2012; Tan et al., 2018; Van Etten et al., 2021).

Mediation models with three mediators were then conducted to evaluate whether and how differences in cortical volumes were mediated by Hcy-related subcortical volumetric differences associated with WMH lesions to influence cognitive aging. In these models, selected cognitive outcomes in three domains—processing speed, executive functioning, and verbal memory—were chosen to limit Type 1 error across multiple serial mediation models and were separately included as dependent variables. Total WMH volume, the Hcy-SGM pattern, and lobar GMVs were tested sequentially as mediators, using the same covariates as above, but additional adjustments were made for the time interval between MRI scans and neuropsychological test administration (Mean ± *SD* = 58.56 ± 47.35 days) to control for the potential influence of differences in time lengths between assessments on the outcome variables (Habes et al., 2016). In a follow-up analysis, we additionally included depression ratings on the Geriatric Depression Scale (Yesavage et al., 1982) as an added covariate to control for its potential relation to cognitive performance.

We applied a bootstrapping procedure with 10,000 iterations in the mediation analyses using the PROCESS macro v3.5 (Hayes, 2018) to construct 95% percentile confidence intervals (CIs) for indirect effects. Significant mediation effects were determined by the percentile bootstrap CIs that did not contain zero. Completely standardized indirect effects were reported as effect size measures (Preacher and Kelley, 2011). All statistical analyses were performed using SPSS 28.0 (IBM Corp., Armonk, NY).

## 3 Results

There was a significant inverse association of the Hcy-SGM network pattern characterized by hippocampal and nucleus accumbens volume reductions and relative volume preservation in basal ganglia regions with parietal lobe GMV (*β* = −0.188, *p-*FDR *=* 0.0017) after we controlled for TIV, age, sex, and education. While a trend toward an inverse association was observed for frontal gray matter (*β* = −0.116, *p-*FDR *=* 0.0510), the Hcy-SGM pattern did not show significant associations with each of the temporal and occipital lobe GMVs after adjustments for the same covariates with or without multiple comparison correction (all *p*’s > 0.05; Table 2). When these analyses were repeated with hemispheric lobar cortical volumes separately as outcome variables, while controlling for the same covariates listed above, the results were consistent across hemispheres (see Supplementary Table 1).

**Table 2:** Summary of multiple regression analyses for the Hcy-SGM network pattern predicting lobar regions of cortical brain volume.

Variable	*β*	*B*	*SE*	95% CI for *B*	*p*-value	*p-*FDR^a^
Frontal GMV	−0.116	−1.742	0.772	−3.267, −0.216	0.0255	0.0510
Temporal GMV	−0.096	−0.920	0.514	−1.936, 0.095	0.0754	0.1005
Parietal GMV	−0.188	−2.106	0.585	−3.260, −0.951	0.0004	0.0017
Occipital GMV	−0.092	−0.502	0.381	−1.255, 0.251	0.1898	0.1898

*β* represents the standardized coefficient. *B* and *SE* indicate the unstandardized coefficient and standard error of *B*, respectively, with adjustments for TIV, age, sex, and years of education.

a *p*-value adjusted for multiple comparisons.

CI, confidence interval; GMV, gray matter volume; Hcy, homocysteine; SGM, subcortical gray matter; TIV, total intracranial volume.

**Figure 1:**
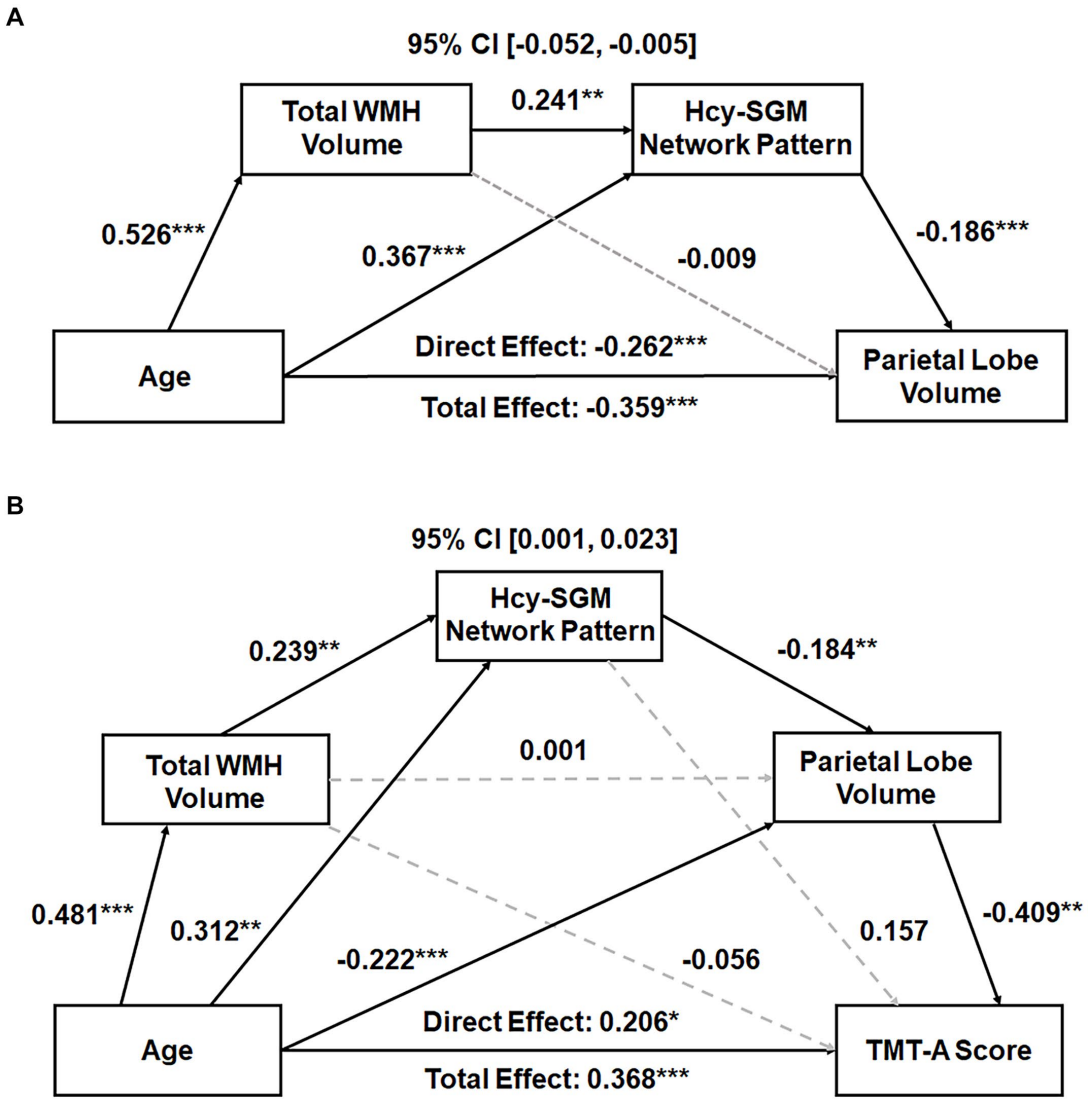
**(A)** Total WMH volume and the Hcy-related SGM network SSM pattern sequentially mediating the association between age and parietal GMV. Standardized path coefficients with TIV, sex, and years of education as covariates are presented. The mediation effects remained significant after additional adjustments for APOE ε4 status, hypertension status, smoking history, VO_2_max, and further vitamin B12 levels. **(B)** Total WMH volume, Hcy-related SGM network pattern, and parietal GMV sequentially mediating the relationship between age and processing speed performance. Standardized path coefficients while controlling for TIV, sex, years of education, the time interval between MRI scans and neuropsychological tests, APOE ε4 status, hypertension status, smoking history, VO_2_max, and additionally vitamin B12 levels as covariates are presented. This serial mediation effect remained significant after additional adjustment for depression ratings. Boxes and paths indicate hypothesized variables and their associations. Black solid and gray dotted lines indicate statistically significant and non-significant paths, respectively. In these models, zero did not lie within the 95% CIs, indicating a significant sequential mediation effect. APOE, apolipoprotein E; CI, confidence interval; GMV, gray matter volume; Hcy, homocysteine; SGM, subcortical gray matter; SSM, Scaled Subprofile Model; TIV, total intracranial volume; TMT, Trail Making Test; VO_2_max, volume of maximal oxygen consumption; WMH, white matter hyperintensity. *p < 0.05, **p < 0.01, ***p < 0.001.

Subsequently, serial mediation models revealed, in addition to significant direct effects, significant indirect effects of age sequentially through WMH volume and the Hcy-SGM pattern on parietal lobe GMV (Effect = −0.024, *SE* = 0.012, 95% CI [−0.052; −0.005]) while accounting for differences in TIV, sex, and education. Individual path associations indicated that increasing age predicted greater global WMH burden (*β* = 0.526, *p* < 0.0001), followed by greater pattern expression (*β* = 0.241, *p* = 0.0037), which then predicted smaller parietal lobe GMV (*β* = −0.186, *p* = 0.0007; Figure 1A). The mediation effects remained significant after additional adjustments for APOE ε4 status, hypertension status, smoking history, VO_2_max, and further vitamin B12 levels (Effect = −0.020, *SE* = 0.012, 95% CI [−0.048; −0.003]).

We additionally performed a sensitivity analysis to test an alternative mediation model where the brain imaging mediators (WMH lesion load and the Hcy-SGM pattern) were reversed in order, to establish whether our proposed hypothesized sequence was specifically supported. The indirect effect for this reversed order model was not significant in the fully adjusted mediation analysis (Effect = −0.002, *SE* = 0.005, 95% CI [−0.012; 0.009]), further supporting our proposed model. In addition, follow-up linear regressions to further evaluate the relation between Hcy and other common cardiovascular risk factors revealed that there were non-significant associations of Hcy with each of the vascular risk factors included as covariates in our models with or without adjusting for demographics (all *p*’s > 0.05; see Supplementary Table 2).

We then included cognitive outcomes in the mediation analysis to examine whether and how lobar regions of cortical brain volume impact cognitive aging, while focusing on three selected cognitive measures. There were no significant indirect effects of age via WMH lesion load, the Hcy-SGM pattern, and parietal GMV for the SRT CLTR score (Effect = −0.005, *SE* = 0.005, 95% CI [−0.017;0.001]) and for the residualized TMT-B value (Effect = 0.001, *SE* = 0.005, 95% CI [−0.009;0.011]), while controlling for TIV, sex, education, and the time interval between MRI scans and neuropsychological tests (Table 3).

**Table 3:** Total, direct, and indirect effects of age predicting cognitive function sequentially through WMH volume, the Hcy-SGM network pattern, and parietal GMV.

Cognitive measures	Indirect effects			Direct effects	Total effects
	Effect	*SE*	LLCI, ULCI	Effect	Effect
SRT CLTR	−0.005	0.005	−0.017, 0.001	−0.428^***^	−0.436^***^
TMT-B^a^	0.001	0.005	−0.009, 0.011	0.175	0.165^*^
TMT-A^b^	0.010	0.006	0.001, 0.024	0.246^**^	0.445^***^

Effect and *SE* represent standardized coefficients and standard error, respectively, with TIV, sex, years of education, and the time interval between MRI scans and neuropsychological tests as covariates. LLCI and ULCI represent lower and upper bounds of a 95% bootstrap CI for the indirect effect. That zero does not lie within the 95% CIs indicates a significant mediation effect.

a Standardized residual value of TMT-B obtained by statistically removing the processing speed performance on TMT-A.

b Log transformed value.

CI, confidence interval; CLTR, consistent long-term retrieval; GMV, gray matter volume; Hcy, homocysteine; SGM, subcortical gray matter; SRT, Buschke Selective Reminding Test; TIV, total intracranial volume; TMT, Trail Making Test; WMH, white matter hyperintensity.

^*^p < 0.05, ^**^p < 0.01, ^***^p < 0.001.

With the same covariates listed above, however, a significant indirect effect sequentially through WMH burden, the Hcy-SGM pattern, and volume of parietal gray matter was observed in the association between age and visuomotor processing speed (Effect = 0.010, *SE* = 0.006, 95% CI [0.001;0.024]), along with significant direct age effects (Table 3). These indirect effects for processing speed remained significant after additionally controlling for APOE ε4 status, hypertension status, smoking history, VO_2_max, and vitamin B12 levels (Effect = 0.009, *SE* = 0.006, 95% CI [0.001;0.023]; Figure 1B) and were not attenuated after adjustment for depression ratings (Effect = 0.010, *SE* = 0.006, 95% CI [0.001;0.026]). As such, increasing age predicted greater total WMH volume (*β* = 0.481, *p* < 0.0001), which predicted greater expression of the Hcy-SGM network pattern (*β* = 0.239, *p* = 0.0069), which then predicted smaller parietal GMV (*β* = −0.184, *p* = 0.0010), followed by slowed processing speed (*β* = −0.409, *p* = 0.0018).

## 4 Discussion

In this cohort of healthy older adults, we found smaller parietal lobe volumes associated with greater Hcy-SGM network pattern expression, which was associated with increased WMH burden. We further found in addition to direct effects of age on processing speed, indirect effects through WMH lesion load, the Hcy-SGM pattern, and then parietal lobe atrophy, suggesting that slowed processing speed in cognitive aging may be partly attributable to these brain imaging markers. These findings from the present study expand upon our prior work that has shown associations of a multivariate Hcy-SGM pattern characterized by Hcy-related volume reductions in both hippocampus and nucleus accumbens with relative preservation in basal ganglia with cognitive functioning, by evaluating the pattern’s association with cortical lobar brain volumes. In addition, we assessed the mediating roles of these brain imaging markers to identify a possible vascular brain pathway associated with cognitive dysfunction in healthy aging.

There was an inverse association between the Hcy-SGM pattern and volumes of parietal gray matter consistent across hemispheres, which was not influenced by differences in TIV, age, sex, and education, suggesting parietal brain atrophy associated with cortical impacts of Hcy in combination with its subcortical brain-based effects (Song et al., 2023). These findings are consistent with the existing literature that has reported reductions in cortical gray matter of the parietal lobe related to higher Hcy levels in non-demented older adults even when controlling for other vascular risk factors (Tan et al., 2018).

Studies in animal models have linked glutamatergic neurotoxic effects of Hcy to brain atrophy in parietal cortex, as well as the hippocampus (Lipton et al., 1997; Matté et al., 2009, 2010; MacHado et al., 2011), which might be facilitated by breakdown of the blood–brain barrier and increased permeability of brain micro-vessels induced by Hcy (Kamath et al., 2006), resulting in apoptotic and excitotoxic cell death in these brain areas. Prior studies have additionally suggested that vascular pathology related to elevated Hcy concentrations may be associated with brain atrophy, given the pro-atherothrombotic effects of Hcy, producing inflammation and endothelial dysfunction in cerebral blood vessels (Hankey and Eikelboom, 2001; Faraci and Lentz, 2004). Studies of middle-to-older-aged adults have also shown a relationship between Hcy and WMH lesion load (Wright et al., 2005; Raz et al., 2012).

In line with these studies, our results showed an association of increased WMH burden related to aging with smaller parietal GMV, through greater Hcy-SGM pattern expression. This sequence of associations in our proposed model was further confirmed in a follow-up sensitivity analysis using an alternative order of predictors in the mediation model which showed no significant indirect effects, indicating the robustness of our primary model results. These findings are consistent with previous studies reporting parietal lobe atrophy related to greater WMH volume (Raji et al., 2012; Lambert et al., 2015; Habes et al., 2016), but also extend previous findings by showing its indirect effects via reduced hippocampal and nucleus accumbens volumes with Hcy in the context of healthy aging. Notably, the direct effects of age on parietal GMV remained significant, indicating that the sequential brain imaging mediators—WMH lesion load and the Hcy-SGM pattern—partially explain the influence of age on parietal GMV.

It is possible that elevated Hcy may lead to morphological differences in SGM through vascular pathology, promoting vulnerability of parietal cortical brain volume by vascular impacts of WMH lesion load related to ischemic damage (Du et al., 2005; Erten-Lyons et al., 2013). Another possible explanation may be axonal damage associated with WMH burden that disrupts cortico-subcortical connections, particularly to the hippocampus and nucleus accumbens (Du et al., 2005; Gouw et al., 2011; Kravitz et al., 2011; Britt et al., 2012; Erten-Lyons et al., 2013), followed by brain atrophy of the involved regions (Schmidt et al., 2005).

The parietal lobe includes brain regions that have shown preferential atrophy in previous studies of mild cognitive impairment and AD, such as in the precuneus, posterior cingulate gyrus, and inferior parietal lobule (Jacobs et al., 2012). These results from prior work suggest the possibility that parietal lobe atrophy associated with both Hcy-related subcortical morphological differences and WMH burden represents a potential vascular linkage to increased AD risk (Hooshmand et al., 2013; Habes et al., 2016). Future research, however, is needed to further examine region-specific vulnerability to Hcy-and WMH-related cumulative vascular burden of the aging brain. It would also be important to evaluate the potential role of parietal lobe atrophy with decreased SGM volumes of both the hippocampus and nucleus accumbens by Hcy in dementia risk related to CVD and its implications for the common association of vascular risk factors in the development of AD during healthy aging.

When serial mediation models were extended to include cognition, we also found that greater WMH volume related to aging was associated with greater expression of the Hcy-SGM pattern, followed by parietal lobe atrophy and then slowed processing speed. That such indirect effects remained significant after adjusting for AD-related and other vascular health risk factors, as well as vitamin B12 levels and depressive symptoms, indicates the robustness of our observed findings. These findings are consistent with the literature on the association between elevated Hcy and reductions in cortical and/or subcortical gray matter, and diminished processing speed even after controlling for other vascular risk factors (Dufouil et al., 2003; Seshadri et al., 2008; Tan et al., 2018; Song et al., 2023). We also observed that Hcy was not significantly associated with the other common vascular risk factors included as covariates in our models, including APOE ε4 status, hypertension status, smoking history, and VO_2_max. Together, these findings suggest that Hcy and other common cardiovascular risk factors may have separable or additive effects on brain structure and cognition. Future work is needed to further evaluate how the Hcy-related SGM pattern is influenced by other clinical and vascular health risk factors.

In addition, these results are in accord with prior research that has linked high plasma Hcy levels to cognitive dysfunction, with the relation most pronounced for processing speed (Prins et al., 2002; Dufouil et al., 2003; Feng et al., 2013), but are also consistent with studies showing preferential associations between CVD and slowed processing speed (Turken et al., 2008; Jacobs et al., 2013; Righart et al., 2013). Deficits in processing speed have been shown to be a leading indicator of age-related differences in cognition (Salthouse, 1996), and to predict progression from being cognitively unimpaired to mild cognitive impairment in a recent study (Rabin et al., 2020). Our results suggest linkages between reduced parietal GMV associated with Hcy-related subcortical volumetric differences, WMH load, and an important aspect of cognitive aging.

Although white matter tracts were not directly assessed in the present study, slowing of processing speed related to vascular white matter damage has been associated with a disruption of fronto-subcortical circuits driven by ischemic lesions and the associated alterations in cortical brain structures (Righart et al., 2013). A key role of frontoparietal white matter pathways in age-related differences in processing speed performance, however, has also been suggested in diffusion tensor imaging studies of non-demented older adults (Salami et al., 2012; Jacobs et al., 2013). In support of this association, a study on CVD reported that cortical lesions in parietal brain regions, including supramarginal and angular gyri, together with parietal lobe white matter lesions were associated with diminished processing speed (Turken et al., 2008).

Additionally, converging evidence from non-human primate and human studies has suggested that some of the parietal lobe regions and the hippocampus are part of a parieto-medial temporal pathway involved in attentional and visuospatial processing (Kravitz et al., 2011). It has also been shown in a recent human neuroimaging study that nucleus accumbens is involved in encoding of effortful information processing (Suzuki et al., 2021). Taken together, these findings suggest a vascular risk pathway where white matter damage associated with WMH lesions may contribute to subcortical and cortical brain-based impacts, influenced by Hcy, which may drive a disruption of frontal–parietal-subcortical connections, promoting volume reductions in the involved brain regions, which may in turn be associated with slowed processing speed (Turken et al., 2008; Kravitz et al., 2011; Jacobs et al., 2013; Bennett and Madden, 2014; Suzuki et al., 2021).

Despite associations shown in prior literature between reduced cortical gray matter and diminished executive functioning and memory in aging (Cardenas et al., 2011; Mungas et al., 2018), we did not find relations of parietal lobe atrophy associated with the Hcy-SGM pattern, as well as WMH burden, to these cognitive domains, which was consistent with our prior work (Song et al., 2023). Other previous studies, however, have also shown significant associations of Hcy with processing speed but not with executive functioning or memory after controlling for sex, education, and other vascular risk factors (Dufouil et al., 2003; Feng et al., 2013). These findings suggest that certain cognitive domains may be differentially vulnerable to WMH lesions and cortical and subcortical brain-based impacts of Hcy (Gunning-Dixon and Raz, 2000; Dufouil et al., 2003; Prins et al., 2005; Feng et al., 2013). It is also possible that these brain imaging markers may be related to other age-sensitive cognitive domains via their associations with processing speed. A central role for diminished processing speed has been suggested in explaining cross-sectional age-related differences in executive functioning and memory (Salthouse, 1996). It could also be that performance on tests that load primarily on other aspects of executive function may be more sensitive to the structural brain-based impacts of Hcy (Prins et al., 2002).

In addition, previous research has reported an association between smaller frontal and temporal GMV and higher Hcy levels in healthy middle-aged adults (Seshadri et al., 2008). We observed only a non-significant trend towards an inverse relationship between the Hcy-related network pattern of decreased SGM volume and frontal lobe volume after multiple comparison correction. These findings in our study could be attributed to differences in confounding factors controlled for in our models, the study design (longitudinal or cross-sectional), and the size and composition of the study samples, as well as differences in health status, including a relatively lower prevalence of vascular health risk factors in our cohort. Notably, the current study focused on predominantly healthy older adults who were able to perform a maximal treadmill GXT safely, indicating overall good cardiovascular health of our sample. While the healthy older adult cohort is one of the current study’s strengths, the relatively small sample size may limit the ability to detect smaller effects, as well as the generalizability of our findings. These factors, individually or in combination, might influence the strengths of associations in some key findings.

Future work with larger and more ethnically diverse samples and with greater vascular health burden is needed to evaluate the possibility of increased vulnerability of parietal gray matter to structural brain-based effects of Hcy and to assess the generalizability of our findings. It is possible that associations of Hcy-related subcortical volumetric differences with frontal and temporal gray matter might be observed in future work with larger and more diverse samples. It is also possible that associations of greater parietal lobe atrophy with decrements in executive functions and memory might be observed in larger cohorts.

Using serial mediation models with bootstrap resampling, we tested a hypothesized pathway that showed significant, robust indirect effects of differences in brain imaging markers in predicting a key aspect of cognitive aging. Combined with the findings of partial mediation in the present study, future research would be important to evaluate other factors, such as inflammation-related genetic variants, that might influence the observed associations that were not accounted for in our analyses but could be related to either WMH lesion load and Hcy-related brain atrophy or cognitive functioning or both.

Moreover, the relationships between WMH volume, Hcy-related subcortical volumetric differences, and cognitive functioning were assessed cross-sectionally in the present study. Although our hypothesized pathway is theoretically supported, it is important to note that the cross-sectional design does not permit conclusions regarding causality. Intervention and longitudinal neuroimaging studies investigating Hcy-related subcortical and cortical volumetric changes associated with WMH lesions to evaluate their influence on slowed processing speed and subsequent progression to cognitive impairment in aging are warranted to further evaluate the directionality implied by the current results. Furthermore, future longitudinal work with larger samples would be important in clarifying the causal relationships and implications of our findings for the early development of vascular cognitive impairment, as well as potential linkages to the risk for AD.

## 5 Conclusion

Together, our results indicate that parietal lobe atrophy associated with a multivariate Hcy-related SGM pattern characterized by reduced volumes of both the hippocampus and nucleus accumbens during aging may provide an important early indicator of slowed processing speed in cognitive aging. These findings may reflect a potential link between a common peripheral plasma biomarker of cardiovascular risk, Hcy, CVD, and cognitive dysfunction in healthy aging.

## Data availability statement

The raw data supporting the conclusions of this article will be made available by the authors, without undue reservation.

## Ethics statement

This study involving humans was approved by the University of Arizona IRB committee. The study was conducted in accordance with the local legislation and institutional requirements. The participants provided their written informed consent to participate in this study.

## Author contributions

HS: Conceptualization, Formal analysis, Methodology, Writing – original draft. PB: Writing – review & editing, Formal analysis. DR: Investigation, Methodology, Writing – review & editing. CH: Formal analysis, Software, Writing – review & editing. MG: Investigation, Writing – review & editing. MH: Investigation, Project administration, Writing – review & editing. GH: Investigation, Project administration, Writing – review & editing. TT: Investigation, Project administration, Writing – review & editing. GA: Conceptualization, Funding acquisition, Investigation, Methodology, Project administration, Supervision, Writing – review & editing.

## Footnotes

^1^ http://surfer.nmr.mgh.harvard.edu

^2^ www.itksnap.org

## References

[ref1] ACSM (2000). American College of Sports Medicine (ACSM’s) guidelines for exercise testing and prescription. 6th Edn. Baltimore, MD: Lippincott Williams and Wilkins.

[ref2] Addya K. Wang Y. L. Leonard D. G. (1997). Optimization of apolipoprotein e genotyping. Mol. Diagnosis 2, 271–276. doi: 10.1016/S1084-8592(97)80038-0 10462619

[ref3] Alexander G. E. Bergfield K. L. Chen K. Reiman E. M. Hanson K. D. Lin L.. (2012a). Gray matter network associated with risk for Alzheimer’s disease in young to middle-aged adults. Neurobiol. Aging 33, 2723–2732. doi: 10.1016/j.neurobiolaging.2012.01.014, PMID: 22405043 PMC3398228

[ref4] Alexander G. E. Chen K. Merkley T. L. Reiman E. M. Caselli R. J. Aschenbrenner M.. (2006). Regional network of magnetic resonance imaging gray matter volume in healthy aging. Neuroreport 17, 951–956. doi: 10.1097/01.wnr.0000220135.16844.b6 16791083

[ref5] Alexander G. E. Moeller J. R. (1994). Application of the scaled subprofile model to functional imaging in neuropsychiatric disorders: a principal component approach to modeling brain function in disease. Hum. Brain Mapp. 2, 79–94. doi: 10.1002/hbm.460020108

[ref6] Alexander G. E. Ryan L. Bowers D. Foster T. C. Bizon J. L. Geldmacher D. S.. (2012b). Characterizing cognitive aging in humans with links to animal models. Front. Aging Neurosci. 4:21. doi: 10.3389/fnagi.2012.00021, PMID: 22988439 PMC3439638

[ref7] Benjamini Y. (2010). Discovering the false discovery rate. J. R. Stat. Soc. Ser. B Stat Methodol. 72, 405–416. doi: 10.1111/j.1467-9868.2010.00746.x

[ref8] Benjamini Y. Hochberg Y. (1995). Controlling the false discovery rate: a practical and powerful approach to multiple testing. J. R. Stat. Soc. Ser. B 57, 289–300. doi: 10.1111/j.2517-6161.1995.tb02031.x

[ref9] Bennett I. J. Madden D. J. (2014). Disconnected aging: cerebral white matter integrity and age-related differences in cognition. Neuroscience 276, 187–205. doi: 10.1016/j.neuroscience.2013.11.026, PMID: 24280637 PMC4032380

[ref10] Berry M. J. Brubaker P. H. O’Toole M. L. Rejeski W. J. Soberman J. Ribisl P. M.. (1996). Estimation of V̇O2 in older individuals with osteoarthritis of the knee and cardiovascular disease. Med. Sci. Sports Exerc. 28, 808–814. doi: 10.1097/00005768-199607000-00006, PMID: 8832533

[ref11] Britt J. P. Benaliouad F. McDevitt R. A. Stuber G. D. Wise R. A. Bonci A. (2012). Synaptic and Behavioral profile of multiple glutamatergic inputs to the nucleus Accumbens. Neuron 76, 790–803. doi: 10.1016/j.neuron.2012.09.040, PMID: 23177963 PMC3607383

[ref12] Burnham K. P. Anderson D. R. (2002). Model selection and multimodel inference: A practical information-theoretic approach. 2nd Edn. New York, NY: Springer.

[ref13] Buschke H. (1973). Selective reminding for analysis of memory and learning. J. Verbal Learning Verbal Behav. 12, 543–550. doi: 10.1016/S0022-5371(73)80034-9

[ref14] Cardenas V. A. Chao L. L. Studholme C. Yaffe K. Miller B. L. Madison C.. (2011). Brain atrophy associated with baseline and longitudinal measures of cognition. Neurobiol. Aging 32, 572–580. doi: 10.1016/j.neurobiolaging.2009.04.011, PMID: 19446370 PMC2891686

[ref15] Chao L. L. Mohlenhoff B. S. Weiner M. W. Neylan T. C. (2014). Associations between subjective sleep quality and brain volume in gulf war veterans. Sleep 37, 445–452. doi: 10.5665/sleep.3472 24587566 PMC3920309

[ref16] Desikan R. S. Ségonne F. Fischl B. Quinn B. T. Dickerson B. C. Blacker D.. (2006). An automated labeling system for subdividing the human cerebral cortex on MRI scans into gyral based regions of interest. NeuroImage 31, 968–980. doi: 10.1016/j.neuroimage.2006.01.021, PMID: 16530430

[ref17] Dong C. Nabizadeh N. Caunca M. Cheung Y. K. Rundek T. Elkind M. S. V.. (2015). Cognitive correlates of white matter lesion load and brain atrophy. Neurology 85, 441–449. doi: 10.1212/WNL.0000000000001716, PMID: 26156514 PMC4534076

[ref18] Du A. T. Schuff N. Chao L. L. Kornak J. Ezekiel F. Jagust W. J.. (2005). White matter lesions are associated with cortical atrophy more than entorhinal and hippocampal atrophy. Neurobiol. Aging 26, 553–559. doi: 10.1016/j.neurobiolaging.2004.05.002, PMID: 15653183

[ref19] Dufouil C. Alpérovitch A. Ducros V. Tzourio C. (2003). Homocysteine, white matter hyperintensities, and cognition in healthy elderly people. Ann. Neurol. 53, 214–221. doi: 10.1002/ana.10440, PMID: 12557288

[ref20] Efron B. T. Tibshirani R. J. (1994). An introduction to the bootstrap. Boca Raton, FL: CRC Press.

[ref21] Erten-Lyons D. Woltjer R. Kaye J. Mattek N. Dodge H. H. Green S.. (2013). Neuropathologic basis of white matter hyperintensity accumulation with advanced age. Neurology 81, 977–983. doi: 10.1212/WNL.0b013e3182a43e45, PMID: 23935177 PMC3888199

[ref22] Faraci F. M. Lentz S. R. (2004). Hyperhomocysteinemia, oxidative stress, and cerebral vascular dysfunction. Stroke 35, 345–347. doi: 10.1161/01.STR.0000115161.10646.67 14757874

[ref23] Feng L. Isaac V. Sim S. Ng T. P. Krishnan K. R. R. Chee M. W. L. (2013). Associations between elevated homocysteine, cognitive impairment, and reduced white matter volume in healthy old adults. Am. J. Geriatr. Psychiatry 21, 164–172. doi: 10.1016/j.jagp.2012.10.017, PMID: 23343490

[ref24] Ferris J. Greeley B. Yeganeh N. M. Rinat S. Ramirez J. Black S.. (2022). Exploring biomarkers of processing speed and executive function: the role of the anterior thalamic radiations. NeuroImage Clin. 36:103174. doi: 10.1016/j.nicl.2022.103174, PMID: 36067614 PMC9460835

[ref25] Fischl B. Salat D. H. Busa E. Albert M. Dieterich M. Haselgrove C.. (2002). Whole brain segmentation: automated labeling of neuroanatomical structures in the human brain. Neuron 33, 341–355. doi: 10.1016/S0896-6273(02)00569-X, PMID: 11832223

[ref26] Fischl B. Salat D. H. VanDerKouwe A. J. W. Makris N. Ségonne F. Quinn B. T.. (2004). Sequence-independent segmentation of magnetic resonance images. NeuroImage 23, S69–S84. doi: 10.1016/j.neuroimage.2004.07.016, PMID: 15501102

[ref27] Franchetti M. K. Bharadwaj P. K. Nguyen L. A. VanEtten E. J. Klimentidis Y. C. Hishaw G. A.. (2020). Interaction of age and self-reported physical sports activity on white matter Hyperintensity volume in healthy older adults. Front. Aging Neurosci. 12:346. doi: 10.3389/fnagi.2020.576025, PMID: 33240074 PMC7667263

[ref28] Gouw A. A. Seewann A. Van Der Flier W. M. Barkhof F. Rozemuller A. M. Scheltens P.. (2011). Heterogeneity of small vessel disease: a systematic review of MRI and histopathology correlations. J. Neurol. Neurosurg. Psychiatry 82, 126–135. doi: 10.1136/jnnp.2009.204685, PMID: 20935330

[ref29] Gunning-Dixon F. M. Raz N. (2000). The cognitive correlates of white matter abnormalities in normal aging: a quantitative review. Neuropsychology 14, 224–232. doi: 10.1037/0894-4105.14.2.224, PMID: 10791862

[ref30] Habeck C. Krakauer J. W. Ghez C. Sackeim H. A. Eidelberg D. Stern Y.. (2005). A new approach to spatial covariance modeling of functional brain imaging data: ordinal trend analysis. Neural Comput. 17, 1602–1645. doi: 10.1162/0899766053723023, PMID: 15901409

[ref31] Habes M. Erus G. Toledo J. B. Zhang T. Bryan N. Launer L. J.. (2016). White matter hyperintensities and imaging patterns of brain ageing in the general population. Brain 139, 1164–1179. doi: 10.1093/brain/aww008, PMID: 26912649 PMC5006227

[ref32] Hankey G. J. Eikelboom J. W. (2001). Homocysteine and stroke. Curr. Opin. Neurol. 14, 95–102. doi: 10.1097/00019052-200102000-00015 11176224

[ref33] Hayes A. F. (2018). Introduction to mediation, moderation, and conditional process analysis: A regression-based approach. 2nd Edn. New York, NY: Guilford Publications.

[ref34] Hooshmand B. Polvikoski T. Kivipelto M. Tanskanen M. Myllykangas L. Erkinjuntti T.. (2013). Plasma homocysteine, Alzheimer and cerebrovascular pathology: a population-based autopsy study. Brain 136, 2707–2716. doi: 10.1093/brain/awt206, PMID: 23983028 PMC3754457

[ref35] Jacobs H. I. L. Leritz E. C. Williams V. J. Van Boxtel M. P. J. Elst W. Der J.. (2013). Association between white matter microstructure, executive functions, and processing speed in older adults: the impact of vascular health. Hum. Brain Mapp. 34, 77–95. doi: 10.1002/hbm.21412, PMID: 21954054 PMC3830829

[ref36] Jacobs H. I. L. Van Boxtel M. P. J. Jolles J. Verhey F. R. J. Uylings H. B. M. (2012). Parietal cortex matters in Alzheimer’s disease: an overview of structural, functional and metabolic findings. Neurosci. Biobehav. Rev. 36, 297–309. doi: 10.1016/j.neubiorev.2011.06.009, PMID: 21741401

[ref37] Kamath A. F. Chauhan A. K. Kisucka J. Dole V. S. Loscalzo J. Handy D. E.. (2006). Elevated levels of homocysteine compromise blood-brain barrier integrity in mice. Blood 107, 591–593. doi: 10.1182/blood-2005-06-2506, PMID: 16189268 PMC1895614

[ref38] Kern K. C. Wright C. B. Bergfield K. L. Fitzhugh M. C. Chen K. Moeller J. R.. (2017). Blood pressure control in aging predicts cerebral atrophy related to small-vessel white matter lesions. Front. Aging Neurosci. 9:132. doi: 10.3389/fnagi.2017.00132 28555103 PMC5430031

[ref39] Klein A. Tourville J. (2012). 101 Labeled brain images and a consistent human cortical Labeling protocol. Front. Neurosci. 6:171. doi: 10.3389/fnins.2012.00171, PMID: 23227001 PMC3514540

[ref40] Kravitz D. J. Saleem K. S. Baker C. I. Mishkin M. (2011). A new neural framework for visuospatial processing. Nat. Rev. Neurosci. 12, 217–230. doi: 10.1038/nrn3008, PMID: 21415848 PMC3388718

[ref41] Lambert C. Benjamin P. Zeestraten E. Lawrence A. J. Barrick T. R. Markus H. S. (2016). Longitudinal patterns of leukoaraiosis and brain atrophy in symptomatic small vessel disease. Brain 139, 1136–1151. doi: 10.1093/brain/aww009, PMID: 26936939 PMC4806220

[ref42] Lambert C. Sam Narean J. Benjamin P. Zeestraten E. Barrick T. R. Markus H. S. (2015). Characterising the grey matter correlates of leukoaraiosis in cerebral small vessel disease. NeuroImage Clin. 9, 194–205. doi: 10.1016/j.nicl.2015.07.002, PMID: 26448913 PMC4564392

[ref43] Lipton S. A. Kim W. K. Choi Y. B. Kumar S. D’Emilia D. M. Rayudu P. V.. (1997). Neurotoxicity associated with dual actions of homocysteine at the N-methyl-D-aspartate receptor. Proc. Natl. Acad. Sci. USA 94, 5923–5928. doi: 10.1073/pnas.94.11.5923, PMID: 9159176 PMC20882

[ref44] MacHado F. R. Ferreira A. G. K. Da Cunha A. A. Tagliari B. Mussulini B. H. M. Wofchuk S.. (2011). Homocysteine alters glutamate uptake and Na+,K +-ATPase activity and oxidative status in rats hippocampus: protection by vitamin C. Metab. Brain Dis. 26, 61–67. doi: 10.1007/s11011-011-9232-3 21287399

[ref45] Matté C. Mackedanz V. Stefanello F. M. Scherer E. B. S. Andreazza A. C. Zanotto C.. (2009). Chronic hyperhomocysteinemia alters antioxidant defenses and increases DNA damage in brain and blood of rats: protective effect of folic acid. Neurochem. Int. 54, 7–13. doi: 10.1016/j.neuint.2008.08.011, PMID: 18983880

[ref46] Matté C. Mussulini B. H. M. dos Santos T. M. Soares F. M. S. Simão F. Matté A.. (2010). Hyperhomocysteinemia reduces glutamate uptake in parietal cortex of rats. Int. J. Dev. Neurosci. 28, 183–187. doi: 10.1016/j.ijdevneu.2009.11.004, PMID: 19913086

[ref47] Morys F. Dadar M. Dagher A. (2021). Association between midlife obesity and its metabolic consequences, cerebrovascular disease, and cognitive decline. J. Clin. Endocrinol. Metab. 106, e4260–e4274. doi: 10.1210/clinem/dgab135, PMID: 33677592 PMC8475210

[ref48] Mungas D. Gavett B. Fletcher E. Farias S. T. DeCarli C. Reed B. (2018). Education amplifies brain atrophy effect on cognitive decline: implications for cognitive reserve. Neurobiol. Aging 68, 142–150. doi: 10.1016/j.neurobiolaging.2018.04.002, PMID: 29798764 PMC5993638

[ref49] Pini L. Pievani M. Bocchetta M. Altomare D. Bosco P. Cavedo E.. (2016). Brain atrophy in Alzheimer’s disease and aging. Ageing Res. Rev. 30, 25–48. doi: 10.1016/j.arr.2016.01.002 26827786

[ref50] Preacher K. J. Kelley K. (2011). Supplemental material for effect size measures for mediation models: quantitative strategies for communicating indirect effects. Psychol. Methods 16, 93–115. doi: 10.1037/a0022658.supp 21500915

[ref51] Prins N. D. Den Heijer T. Hofman A. Koudstaal P. J. Jolles J. Clarke R.. (2002). Homocysteine and cognitive function in the elderly: the Rotterdam scan study. Neurology 59, 1375–1380. doi: 10.1212/01.WNL.0000032494.05619.93 12427887

[ref52] Prins N. D. Van Dijk E. J. Den Heijer T. Vermeer S. E. Jolles J. Koudstaal P. J.. (2005). Cerebral small-vessel disease and decline in information processing speed, executive function and memory. Brain 128, 2034–2041. doi: 10.1093/brain/awh553 15947059

[ref53] Rabin J. S. Neal T. E. Nierle H. E. Sikkes S. A. M. Buckley R. F. Amariglio R. E.. (2020). Multiple markers contribute to risk of progression from normal to mild cognitive impairment. NeuroImage Clin. 28:102400. doi: 10.1016/j.nicl.2020.102400, PMID: 32919366 PMC7491146

[ref54] Raji C. A. Lopez O. L. Kuller L. H. Carmichael O. T. Longstreth W. T. Gach H. M.. (2012). White matter lesions and brain gray matter volume in cognitively normal elders. Neurobiol. Aging 33, 834.e7–834.e16. doi: 10.1016/j.neurobiolaging.2011.08.010, PMID: 21943959 PMC3248984

[ref55] Raz N. Yang Y. Dahle C. L. Land S. (2012). Volume of white matter hyperintensities in healthy adults: contribution of age, vascular risk factors, and inflammation-related genetic variants. Biochim. Biophys. Acta Mol. basis Dis. 1822, 361–369. doi: 10.1016/j.bbadis.2011.08.007, PMID: 21889590 PMC3245802

[ref56] Reitan R. M. (1958). Validity of the trail making test as an indicator of organic brain damage. Percept. Mot. Skills 8, 271–276. doi: 10.2466/pms.1958.8.3.271

[ref57] Resnick S. M. Pham D. L. Kraut M. A. Zonderman A. B. Davatzikos C. (2003). Longitudinal magnetic resonance imaging studies of older adults: a shrinking brain. J. Neurosci. 23, 3295–3301. doi: 10.1523/jneurosci.23-08-03295.2003, PMID: 12716936 PMC6742337

[ref58] Righart R. Duering M. Gonik M. Jouvent E. Reyes S. Hervé D.. (2013). Impact of regional cortical and subcortical changes on processing speed in cerebral small vessel disease. NeuroImage Clin. 2, 854–861. doi: 10.1016/j.nicl.2013.06.006, PMID: 24179837 PMC3777834

[ref59] Salami A. Eriksson J. Nilsson L. G. Nyberg L. (2012). Age-related white matter microstructural differences partly mediate age-related decline in processing speed but not cognition. Biochim. Biophys. Acta Mol. basis Dis. 1822, 408–415. doi: 10.1016/j.bbadis.2011.09.001, PMID: 21930202

[ref60] Salthouse T. A. (1996). The processing-speed theory of adult age differences in cognition. Psychol. Rev. 103, 403–428. doi: 10.1037/0033-295X.103.3.403 8759042

[ref61] Salthouse T. A. Toth J. Daniels K. Parks C. Pak R. Wolbrette M.. (2000). Effects of aging on efficiency of task switching in a variant of the trail making test. Neuropsychology 14, 102–111. doi: 10.1037/0894-4105.14.1.102, PMID: 10674802

[ref62] Sánchez-Cubillo I. Periáñez J. A. Adrover-Roig D. Rodríguez-Sánchez J. M. Ríos-Lago M. Tirapu J.. (2009). Construct validity of the trail making test: role of task-switching, working memory, inhibition/interference control, and visuomotor abilities. J. Int. Neuropsychol. Soc. 15, 438–450. doi: 10.1017/S1355617709090626 19402930

[ref63] Schmidt P. Gaser C. Arsic M. Buck D. Förschler A. Berthele A.. (2012). An automated tool for detection of FLAIR-hyperintense white-matter lesions in multiple sclerosis. NeuroImage 59, 3774–3783. doi: 10.1016/j.neuroimage.2011.11.032, PMID: 22119648

[ref64] Schmidt R. Ropele S. Enzinger C. Petrovic K. Smith S. Schmidt H.. (2005). White matter lesion progression, brain atrophy, and cognitive decline: the Austrian stroke prevention study. Ann. Neurol. 58, 610–616. doi: 10.1002/ana.20630, PMID: 16178017

[ref65] Seshadri S. Beiser A. Selhub J. Jacques P. F. Rosenberg I. H. D’Agostino R. B.. (2002). Plasma homocysteine as a risk factor for dementia and Alzheimer’s disease. N. Engl. J. Med. 346, 476–483. doi: 10.1056/NEJMoa011613 11844848

[ref66] Seshadri S. Wolf P. A. Beiser A. S. Selhub J. Au R. Jacques P. F.. (2008). Association of plasma total homocysteine levels with subclinical brain injury: cerebral volumes, white matter hyperintensity, and silent brain infarcts at volumetric magnetic resonance imaging in the Framingham offspring study. Arch. Neurol. 65, 642–649. doi: 10.1001/archneur.65.5.642, PMID: 18474741 PMC2700952

[ref67] Song H. Bharadwaj P. K. Raichlen D. A. Habeck C. G. Huentelman M. J. Hishaw G. A.. (2023). Association of homocysteine-related subcortical brain atrophy with white matter lesion volume and cognition in healthy aging. Neurobiol. Aging 121, 129–138. doi: 10.1016/j.neurobiolaging.2022.10.011, PMID: 36436304 PMC10002471

[ref68] Suzuki S. Lawlor V. M. Cooper J. A. Arulpragasam A. R. Treadway M. T. (2021). Distinct regions of the striatum underlying effort, movement initiation and effort discounting. Nat. Hum. Behav. 5, 378–388. doi: 10.1038/s41562-020-00972-y, PMID: 33230282 PMC8555699

[ref69] Tan B. Venketasubramanian N. Vrooman H. Cheng C. Y. Wong T. Y. Ikram M. K.. (2018). Homocysteine and cerebral atrophy: the epidemiology of dementia in Singapore study. J. Alzheimers Dis. 62, 877–885. doi: 10.3233/JAD-170796, PMID: 29480177

[ref70] Turken A. U. Whitfield-Gabrieli S. Bammer R. Baldo J. V. Dronkers N. F. Gabrieli J. D. E. (2008). Cognitive processing speed and the structure of white matter pathways: convergent evidence from normal variation and lesion studies. NeuroImage 42, 1032–1044. doi: 10.1016/j.neuroimage.2008.03.057, PMID: 18602840 PMC2630965

[ref71] Van Etten E. J. Bharadwaj P. K. Hishaw G. A. Huentelman M. J. Trouard T. P. Grilli M. D.. (2021). Influence of regional white matter hyperintensity volume and apolipoprotein E ε4 status on hippocampal volume in healthy older adults. Hippocampus 31, 469–480. doi: 10.1002/hipo.23308, PMID: 33586848 PMC9119498

[ref72] Walhovd K. B. Fjell A. M. Reinvang I. Lundervold A. Dale A. M. Eilertsen D. E.. (2005). Effects of age on volumes of cortex, white matter and subcortical structures. Neurobiol. Aging 26, 1261–1270. doi: 10.1016/j.neurobiolaging.2005.05.020 16005549

[ref73] Werden E. Cumming T. Li Q. Bird L. Veldsman M. Pardoe H. R.. (2017). Structural MRI markers of brain aging early after ischemic stroke. Neurology 89, 116–124. doi: 10.1212/WNL.0000000000004086 28600458 PMC5501937

[ref74] Wright C. B. Paik M. C. Brown T. R. Stabler S. P. Allen R. H. Sacco R. L.. (2005). Total homocysteine is associated with white matter hyperintensity volume: the northern Manhattan study. Stroke 36, 1207–1211. doi: 10.1161/01.STR.0000165923.02318.22, PMID: 15879345 PMC1352322

[ref75] Yesavage J. A. Brink T. L. Rose T. L. Lum O. Huang V. Adey M.. (1982). Development and validation of a geriatric depression screening scale: a preliminary report. J. Psychiatr. Res. 17, 37–49. doi: 10.1016/0022-3956(82)90033-4 7183759

