# Supplementary material for: Cortical lobar volume reductions associated with homocysteine-related subcortical brain atrophy and poorer cognition in healthy aging
Source: Front Aging Neurosci. 2024 Aug 7;16:1406394. doi: 10.3389/fnagi.2024.1406394 (PMC11335513; doi:10.3389/fnagi.2024.1406394)
Supplement: Supplementary file 1 [file Data_Sheet_1.docx]

Supplementary Material

**Cortical lobar volume reductions associated with homocysteine-related subcortical brain atrophy and poorer cognition in healthy aging**

**Hyun Song^1,2^, Pradyumna K. Bharadwaj^1,2^, David A. Raichlen^3^, Christian G. Habeck^4^, Matthew D. Grilli^1,2,5^, Matthew J. Huentelman^2,6,7^, Georg A. Hishaw^5^, Theodore P. Trouard^2,7,8^, Gene E. Alexander^1,2,7,9,10 *^**

^1^Department of Psychology, University of Arizona, Tucson, AZ, USA

^2^Evelyn F. McKnight Brain Institute, University of Arizona, Tucson, AZ, USA

^3^Human and Evolutionary Biology Section, Department of Biological Sciences, University of Southern California, Los Angeles, CA, USA

^4^Cognitive Neuroscience Division, Department of Neurology and Taub Institute, Columbia University, New York, NY, USA

^5^Department of Neurology, University of Arizona, Tucson, AZ, USA

^6^Neurogenomics Division, The Translational Genomics Research Institute (TGen), Phoenix, AZ, USA

^7^Arizona Alzheimer's Consortium, Phoenix, AZ, USA

^8^Department of Biomedical Engineering, University of Arizona, Tucson, AZ, USA

^9^Department of Psychiatry, University of Arizona, Tucson, AZ, USA

^10^Neuroscience and Physiological Sciences Graduate Interdisciplinary Programs, University of Arizona, Tucson, AZ, USA

*** Correspondence:**

Gene E. Alexander, PhD

[gene.alexander@arizona.edu](mailto:gene.alexander@arizona.edu)

Consistent with the primary results, there was a significant negative association of the Hcy-related SGM network pattern with each of the bilateral parietal GMVs (*β* = -0.170, *p-*FDR *=* 0.0061 for left; *β* = -0.200, *p-*FDR *=* 0.0034 for right); and a trend towards a negative association with each of the bilateral frontal GMVs (*β* = -0.110, *p-*FDR *=* 0.0778 for left; *β* = -0.120, *p-*FDR *=* 0.0607 for right). Additionally, no significant relationships were observed between the Hcy-SGM network pattern and each of left and right temporal GMVs (*β* = -0.093, *p-*FDR *=* 0.1285 for left; *β* = -0.094, *p-*FDR *=* 0.1285 for right) and each of left and right occipital GMVs (*β* = -0.064, *p-*FDR *=* 0.3734 for left; *β* = -0.113, *p-*FDR *=* 0.1351 for right; see **Supplementary Table 1**).

**Supplementary Table 1.** Summary of multiple regression analyses for the Hcy-SGM network pattern predicting hemispheric lobar regions of cortical brain volume

| Variable | *β* | *B* | *SE* | 95% CI for *B* | *p* | *p-*FDR^a^ |
| --- | --- | --- | --- | --- | --- | --- |
| Frontal GMV |  |  |  |  |  |  |
| Left | -0.110 | -0.817 | 0.392 | -1.592, -0.042 | 0.0389 | 0.0778 |
| Right | -0.120 | -0.925 | 0.402 | -1.719, -0.131 | 0.0228 | 0.0607 |
| Temporal GMV |  |  |  |  |  |  |
| Left | -0.093 | -0.489 | 0.290 | -1.061, 0.084 | 0.0937 | 0.1285 |
| Right | -0.094 | -0.431 | 0.258 | -0.941, 0.078 | 0.0964 | 0.1285 |
| Parietal GMV |  |  |  |  |  |  |
| Left | -0.170 | -0.965 | 0.299 | -1.555, -0.375 | 0.0015 | 0.0061 |
| Right | -0.200 | -1.141 | 0.317 | -1.767, -0.515 | 0.0004 | 0.0034 |
| Occipital GMV |  |  |  |  |  |  |
| Left | -0.064 | -0.174 | 0.195 | -0.559, 0.211 | 0.3734 | 0.3734 |
| Right | -0.113 | -0.328 | 0.209 | -0.740, 0.084 | 0.1182 | 0.1351 |

*β* represents the standardized coefficient. *B and SE* indicate the unstandardized coefficient and standard error of *B*, respectively, with adjustments for TIV, age, sex, and years of education. ^a^ *p*-value adjusted for multiple comparisons. CI, confidence interval; GMV, gray matter volume; Hcy, homocysteine; SGM, subcortical gray matter; TIV, total intracranial volume.

We additionally tested the relation of Hcy to the other health risk factors using block-wise regression analysis. Consistent with our previous findings of the non-significant relationship between the SSM Hcy-SGM pattern and these factors, there were non-significant associations of Hcy with APOE ε4 status (*β*=0.035, *p=*0.664; *β*=0.087, *p=*0*.*186), hypertension status (*β*=0.121, *p=*0.154; *β*=0.018, *p=*0.801), smoking history (*β*=0.029, *p=*0.712; *β*=-0.063, *p=*0.348), and VO_2_max (*β*=-0.115, *p=*0.177; *β*=-0.082, *p=*0*.*374) without adjustment (Model 1) or with adjustment for demographic factors (age and sex) and vitamin B12 levels (Model 2), respectively (see **Supplementary Table 2**).

**Supplementary Table 2.** Summary of multiple regression analyses for associations between other vascular health risk factors included in our models as covariates and Hcy

| Variable |  |  | | Model 1^a^ | |  |  |
| --- | --- | --- | --- | --- | --- | --- | --- |
|  | *β* | | *B* | | *SE* | 95% CI for *B* | *p* |
| APOE ε4 status | 0.035 | | 0.020 | | 0.045 | -0.069, 0.108 | 0.664 |
| Hypertension status | 0.121 | | 0.067 | | 0.046 | -0.025, 0.158 | 0.154 |
| Smoking history | 0.029 | | 0.015 | | 0.042 | -0.067, 0.098 | 0.712 |
| VO_2_max | -0.115 | | -0.005 | | 0.004 | -0.013, 0.002 | 0.177 |
|  | | | | | | | |
| Variable |  |  | | Model 2^b^ | |  |  |
|  | *β* | | *B* | | *SE* | 95% CI for *B* | *p* |
| APOE ε4 status | 0.087 | | 0.049 | | 0.037 | -0.024, 0.123 | 0*.*186 |
| Hypertension status | 0.018 | | 0.010 | | 0.040 | -0.068, 0.088 | 0.801 |
| Smoking history | -0.063 | | -0.033 | | 0.035 | -0.102, 0.036 | 0.348 |
| VO_2_max | -0.082 | | -0.004 | | 0.004 | -0.012, 0.004 | 0*.*374 |

*β* represents the standardized coefficient. *B and SE* indicate the unstandardized coefficient and standard error of *B*, respectively. APOE ε4 status, hypertension status, and smoking history were dummy coded 1 for APOE ε4 carriers, self-reported hypertensives, and current or past smokers, respectively.

^a^ Unadjusted.

^b^ Adjusted for age, sex, and vitamin B12 levels.

APOE, apolipoprotein E; Hcy, homocysteine; VO_2_max, volume of maximum oxygen consumption as the index of cardiorespiratory fitness.
